# *Post hoc* Responder and Remission Analyses from Two Studies of SHP465 Mixed Amphetamine Salts Extended-Release Among Adults with Attention-Deficit/Hyperactivity Disorder

**DOI:** 10.1089/cap.2020.0012

**Published:** 2020-08-28

**Authors:** Lenard A. Adler, Brigitte Robertson, Jie Chen, Elias Sarkis

**Affiliations:** ^1^ Department of Psychiatry and NYU Langone Medical Center, New York, New York, USA.; ^2^ Department of Child and Adolescent Psychiatry, NYU Langone Medical Center, New York, New York, USA.; ^3^ Global Clinical Development, Shire, a member of the Takeda group of companies, Lexington, Massachusetts, USA.; ^4^ Biostatistics, Shire, a member of the Takeda group of companies, Lexington, Massachusetts, USA.; ^5^ Sarkis Family Psychiatry, Gainesville, Florida, USA.

**Keywords:** adult, attention-deficit/hyperactivity disorder (ADHD), remission, response, SHP465 mixed amphetamine salts (SHP465 MAS)

## Abstract

***Objectives:*** In two studies of adult attention-deficit/hyperactivity disorder (ADHD), SHP465 mixed amphetamine salts (MAS) extended-release significantly reduced ADHD-Rating Scale, 4th Edition total score (ADHD-RS-IV-TS) versus placebo (PBO). This report describes *post hoc* analyses of SHP465 MAS treatment response and remission rates from those studies.

***Methods:*** Adults with Diagnostic and Statistical Manual of Mental Disorders, 4th Edition, Text Revision–defined ADHD were randomized to SHP465 MAS (12.5–75 mg) or PBO in a 7-week dose-optimization study and to SHP465 MAS (25, 50, or 75 mg) or PBO in a 6-week fixed-dose study. Response was examined using three definitions (definition 1: ≥30% ADHD-RS-IV-TS reduction + Clinical Global Impressions-Improvement [CGI-I] rating of 1 or 2; definition 2: ≥50% ADHD-RS-IV-TS reduction + CGI-I rating of 1 or 2; definition 3: ADHD-RS-IV-TS ≤18). Remission was defined as ADHD-RS-IV-TS ≤12. The Kaplan–Meier analyses assessed time to response or remission.

***Results:*** The intent-to-treat populations included 136 SHP465 MAS and 132 PBO participants in the dose-optimization study and 302 SHP465 MAS and 103 PBO participants in the fixed-dose study. Percentages of participants meeting response criteria (SHP465 MAS vs. PBO) at the final treatment week in the dose-optimization and fixed-dose studies, respectively, were 66.0% versus 31.6% and 72.7% versus 28.3% (definition 1); 47.9% versus 27.6% and 60.6% versus 16.7% (definition 2); and 54.3% versus 30.3% and 52.6% versus 18.3% (definition 3). The remission criterion (SHP465 MAS vs. PBO) at the final treatment week was met by 37.2% versus 19.7% of participants in the dose-optimization study and 39.7% versus 10.0% of participants in the fixed-dose study. Times to response and remission favored SHP465 MAS over PBO in both studies (all nominal log-rank *p* < 0.0001).

***Conclusion:*** These *post hoc* analyses indicate that SHP465 MAS was associated with greater response and remission rates than PBO in adults with ADHD, with times to response and remission also nominally favoring SHP465 MAS.

## Introduction

SHP465 mixed amphetamine salts (SHP465 MAS) extended-release is a once-daily, single-entity MAS product for oral administration approved in the United States for the treatment of attention-deficit/hyperactivity disorder (ADHD) in individuals aged ≥13 years (Mydayis^®^ [mixed salts of a single-entity amphetamine product] 2019). Each SHP465 MAS capsule contains three types of drug-releasing beads (an immediate-release [IR] bead and two different types of delayed-release beads) that contain equal amounts by weight of four salts (dextroamphetamine sulfate, amphetamine sulfate, dextroamphetamine saccharate, and amphetamine aspartate monohydrate), resulting in a 3:1 mixture of dextroamphetamine (*d*-amphetamine)-to-levoamphetamine (*l*-amphetamine) base equivalent.

In three short-term efficacy studies in adults with ADHD, SHP465 MAS produced significantly greater reductions from baseline in ADHD-Rating Scale, 4th Edition total score (ADHD-RS-IV-TS) (Spencer et al. 2008; Frick et al. 2020) and ADHD-RS with adult prompts total score (Weisler et al. 2017) than did placebo (PBO). In addition, the published findings from these studies (Spencer et al. 2008; Weisler et al. 2017; Frick et al. 2020) indicated that the safety and tolerability profile of SHP465 MAS was consistent with that of other long-acting stimulants (Weisler et al. 2006; Spencer et al. 2007; Adler et al. 2008, 2009). Although SHP465 MAS has consistently shown efficacy versus PBO in the treatment of adult ADHD as measured by score reductions on symptom ratings scales, understanding how these rating scale changes relate to overall treatment response and symptomatic remission will further inform clinical judgment related to SHP465 MAS treatment.

Adults diagnosed with ADHD experience functional impairment across a range of domains (Biederman et al. 2006; Murphy and Barkley et al. 2007; de Graaf et al. 2008; Joseph et al. 2018). In the workplace, adults with ADHD hold jobs for shorter periods of time, are fired more frequently, have interpersonal problems with coworkers, and miss more work time (Murphy and Barkley et al. 2007; de Graaf et al. 2008; Joseph et al. 2018). Furthermore, adults with ADHD are more likely to have been arrested, to have an unsatisfactory family life, and to have poor relationships with family members (Biederman et al. 2006). Studies have demonstrated that psychostimulant treatment in adults with ADHD can improve functional outcomes (Buitelaar et al. 2012; Ginsberg et al. 2014; Goodman et al. 2017). However, it is not clear at this time what level of ADHD symptom improvement is associated with improved functional outcomes. To better understand this relationship, it is important to identify clinically relevant definitions of treatment response and ADHD symptom remission.

Multiple definitions have been used to quantify treatment response and remission to ADHD pharmacotherapy (Adler et al. 2009, 2014; Dickson et al. 2011; Jain et al. 2011; Mattingly et al. 2013; Dittmann et al. 2014; Goodman et al. 2017; Weiss et al. 2018, 2019). Definitions of response have generally been based on assessing a specified level of improvement on a symptom rating scale (e.g., a ≥25%–50% reduction on the ADHD-RS) alone or combined with a global measure of improvement, such as a rating of 1 (very much improved) or 2 (much improved) on the Clinical Global Impressions-Improvement (CGI-I) scale (Adler et al. 2009, 2014; Dickson et al. 2011; Jain et al. 2011; Mattingly et al. 2013; Dittmann et al. 2014; Weiss et al. 2018). The use of ADHD-RS-TS reductions as clinically relevant measures of treatment response was examined by Goodman et al. (2010) in an analysis of two studies of lisdexamfetamine in children or adults with ADHD. In that analysis, ADHD-RS-IV-TS reductions of 25%–30% corresponded to a one-point change on the CGI-I (e.g., a transition from minimally improved to improved) and 50%–60% reductions in ADHD-RS-IV-TS were needed to achieve a CGI-I rating of 2 (Goodman et al. 2010).

Although multiple types of remission (e.g., syndromatic, symptomatic, and functional) have been proposed for ADHD (Biederman et al. 2000), there is a lack of consensus and limited guidelines regarding the most appropriate definition (Mattingly et al. 2013). For symptomatic remission, published studies have often operationally defined remission as an ADHD-RS-IV-TS or Adult ADHD Investigator Symptom Rating Scale (AISRS) total score ≤18 points (Dickson et al. 2011; Jain et al. 2011; Mattingly et al. 2013; Goodman et al. 2017; Weiss et al. 2018, 2019). In an analysis of adults, 95% of ADHD controls (i.e., individuals who screened negative for ADHD in a primary care setting) had total scores <24 on the 18-item AISRS (Silverstein et al. 2018), suggesting that this cutoff value on the AISRS could be used as an index of symptomatic remission in adults with ADHD. Although definitions of remission based on ADHD-RS-IV-TS have not been linked to other clinically relevant measures or to functional outcomes, an ADHD-RS-IV-TS <18 is indicative of no substantive ADHD symptoms or ADHD-related impairment because this total score suggests that most item scores do not exceed 1 (i.e., a rating of sometimes) in regard to recent ADHD-related behaviors.

To date, analyses of treatment response and remission with SHP465 MAS in adults with ADHD have not been described. The current report describes *post hoc* analyses of response and remission rates after SHP465 MAS treatment using data from two previously published SHP465 MAS clinical studies (Spencer et al. 2008; Frick et al. 2020). In addition, for the remission analyses, potential factors that may mediate remission (age, sex, and baseline symptom count) were examined.

## Methods

### Study design and treatment

Detailed information regarding the study design and treatment for the two studies included in these analyses has been previously described (Spencer et al. 2008; Frick et al. 2020), and is summarized briefly here. Both study protocols were approved by institutional review boards and conducted in accordance with the World Medical Association Declaration of Helsinki principles.

The first study, hereafter referred to as the dose-optimization study (ClinicalTrials.gov, NCT00150579), was a phase 3, randomized, double-blind, PBO-controlled study in which participants were randomized to SHP465 MAS (12.5–75 mg) or PBO for 7 weeks (Spencer et al. 2008). During dose optimization, participants randomized to SHP465 MAS initiated treatment at 12.5 mg and were titrated to 25 (week 2), 50 (week 3), and 75 mg (week 4) based on efficacy and tolerability. A single dose reduction was permitted from 50 to 37.5 mg at the end of week 3 and from 75 to 62.5 mg at the end of week 4 if these doses were not tolerated. Once an optimized dose was attained (i.e., a dose that was tolerable and produced a ≥30% ADHD-RS-IV-TS decrease from baseline), it was maintained until the end of the study.

The second study, hereafter referred to as the fixed-dose study (ClinicalTrials.gov, NCT00152022), was a phase 3, randomized, PBO-controlled, double-blind study in which participants were randomized to SHP465 MAS (25, 50, or 75 mg) or PBO for 6 weeks (Frick et al. 2020). The 25 mg SHP465 MAS group received 25 mg during weeks 1–6; the 50 mg SHP465 MAS group received 25 mg during week 1, 37.5 mg during week 2, and 50 mg during weeks 3–6; and the 75 mg SHP465 MAS group received 25 mg during week 1, 37.5 mg during week 2, 50 mg during week 3, and 75 mg during weeks 4–6. Modifications to the dosing schedule were not allowed.

### Participants

Both studies included adult men and nonpregnant/nonlactating women (aged 18–55 years) who met Diagnostic and Statistical Manual of Mental Disorders, 4th Edition, Text Revision (DSM-IV-TR; American Psychiatric Association 2000) diagnostic criteria for ADHD. Participants were required to have baseline ADHD-RS-IV-TS ≥24 in the dose-optimization study and ≥32 in the fixed-dose study, and to have had satisfactory medical assessments with no clinically relevant abnormalities based on medical history, physical examinations, or clinical and laboratory evaluations. All participants provided written informed consent before any study-related procedures were conducted. Informed consent documents were written in accordance with Good Clinical Practice Guidelines of the Health Insurance Portability and Accountability Act of 1996.

For both studies, key exclusion criteria included having a comorbid psychiatric diagnosis controlled with a prohibited medication or uncontrolled and associated with clinically relevant symptoms that contraindicate SHP465 MAS use or could confound study assessments; structural cardiac, electrocardiogram, or laboratory anomalies at screening or baseline, a history of hypertension, resting systolic blood pressure >139 mmHg, or resting diastolic blood pressure >89 mmHg; a history (within 6 months before screening) of drug dependence or substance use disorder according to DSM-IV-TR criteria (excluding nicotine); or a documented allergy to, intolerance of, or documented history of nonresponsivity to methylphenidate (MPH) or amphetamines.

### Endpoints

The primary efficacy measure in both studies was the ADHD-RS-IV scale. The ADHD-RS-IV is an 18-item, clinician-administered scale (DuPaul et al. 1998). Severity for each item is scored from 0 (none) to 3 (severe), with total score ranging from 0 to 54. The ADHD-RS-IV can be used to assess adult ADHD symptoms with training and the use of prompts (Adler and Cohen 2004; Murphy and Adler 2004; Adler et al. 2005). In the studies included in these analyses, training on use of the ADHD-RS-IV with adult prompts was conducted using standardized methods. The key secondary efficacy measure in both studies was the CGI-I scale. The CGI-I is an eight-point, clinician-rated scale assessing patient improvement relative to baseline; ratings range from 0 (not assessed) to 7 (very much worse) (Guy 1976). In both studies, the ADHD-RS-IV was assessed at baseline and all on-treatment study visits, and the CGI-I was assessed at all postbaseline on-treatment visits.

### Data presentation and analysis

Findings for the prespecified primary efficacy (change in ADHD-RS-IV-TS from baseline to study endpoint) and key secondary (improvement on the dichotomized CGI-I) analyses have been described previously (Spencer et al. 2008; Frick et al. 2020). The *post hoc* analyses reported here describe response and remission rates in the intent-to-treat (ITT) population (i.e., randomized participants who received ≥1 study drug dose and who had a baseline and ≥1 postbaseline primary efficacy endpoint).

Three definitions of response were used. Of these, two represented variations of previously described definitions (Adler et al. 2009; Jain et al. 2011; Mattingly et al. 2013) and used reductions in ADHD-RS-IV-TS combined with specified ratings on the CGI-I scale (≥30% ADHD-RS-IV-TS reduction + CGI-I rating of 1 or 2 [definition 1]; ≥50% ADHD-RS-IV-TS reduction + CGI-I rating of 1 or 2 [definition 2]). The remaining response definition was based solely on ADHD-RS-IV-TS (ADHD-RS-IV-TS ≤18 [definition 3]), and has previously been used as a definition of remission (Dickson et al. 2011; Jain et al. 2011; Mattingly et al. 2013; Goodman et al. 2017; Weiss et al. 2018, 2019). This definition was used to specify response in the current analyses because a more stringent definition of remission was used.

In the current analyses, the intention was to choose an operational definition of symptomatic remission that was highly conservative. Therefore, the definition of remission used was an ADHD-RS-IV-TS ≤12. This score is lower than the score of 18 used to define remission based on the ADHD-RS in previous reports (Dickson et al. 2011; Jain et al. 2011; Mattingly et al. 2013; Goodman et al. 2017; Weiss et al. 2018, 2019). In addition, an ADHD-RS-IV-TS ≤12 is equivalent to one-half of an AISRS total score of 24. In an analysis of the AISRS by Silverstein et al. (2018), 95% of adult ADHD controls (i.e., adults without ADHD) had AISRS total scores <24. As there are 18 items in the ADHD-RS-IV, a total score ≤12 indicates that most items were likely scored as 0 or 1, which is indicative of a low level of ADHD symptoms and no significant ADHD-related impairment.

All analyses consisted of assessing the percentage of participants in each treatment group meeting the respective response and remission criteria at each postbaseline study visit (all response definitions and remission) and at endpoint (response definition 3 [ADHD-RS-IV-TS ≤18] and remission). Endpoint was defined as the average of the last 3 weeks of treatment or the last postbaseline assessment if the last 3 weeks of treatment were missing. In addition, to examine factors that potentially influence remission, subgroup analyses were conducted at endpoint based on sex (men vs. women), age (greater than/equal to vs. less than the mean age), and baseline symptom count (greater than/equal to vs. less than the mean baseline symptom count). Kaplan–Meier analyses were used to examine time to response or remission. Because the studies were not powered for these *post hoc* analyses, all *p* values are nominal and reported for descriptive purposes only.

## Results

### Participant disposition and demographics

The ITT population of the dose-optimization and fixed-dose studies, respectively, consisted of 268 (SHP465 MAS, *n* = 136; PBO, *n* = 132) and 405 (all SHP465 MAS, *N* = 302 [25 mg, *n* = 103; 50 mg, *n* = 101; 75 mg, *n* = 98]; PBO, *n* = 103) participants. Baseline demographic and clinical characteristics were generally comparable between the SHP465 MAS and PBO treatment groups in both studies (Table 1).

**Table 1.** Participant Demographic and Clinical Characteristics, Intent-to-Treat Population

	Dose-optimization study^a^					
	All SHP465 MAS (*n* = 136)				PBO (*n* = 132)	All (*N* = 268)
Mean ± SD age, years	36.1 ± 10.08				37.1 ± 10.26	36.6 ± 10.16
Female, *n* (%)	67 (49.3)				65 (49.2)	132 (49.3)
Race, *n* (%)						
White	117 (86.0)				110 (83.3)	227 (84.7)
Black/African American	9 (6.6)				12 (9.1)	21 (7.8)
Asian	4 (2.9)				3 (2.3)	7 (2.6)
Other	6 (4.4)				7 (5.3)	13 (4.9)
Mean ± SD weight,^b^ lb	180.8 ± 43.49				178.9 ± 40.90	179.9 ± 42.16
Mean ± SD BMI,^c^ kg/m^2^	27.7 ± 5.40				27.4 ± 5.30	ND
Mean ± SD ADHD-RS-IV-TS	35.7 ± 7.49				36.0 ± 7.44	ND
ADHD subtype, *n* (%)						
Inattentive	37 (27.2)				33 (25.0)	70 (26.1)
Hyperactive/impulsive	4 (2.9)				4 (3.0)	8 (3.0)
Combined	95 (69.9)				95 (72.0)	190 (70.9)
	*Fixed-dose study*					
	*All SHP465 MAS (*n* = 302)*	*25 mg (*n* = 103)*	*50 mg (*n* = 101)*	*75 mg (*n* = 98)*	*PBO (*n* = 103)*	*All (*N* = 405)*
Mean ± SD age, years	37.6 ± 9.68	37.8 ± 9.84	37.2 ± 9.51	38.0 ± 9.77	35.6 ± 9.82	37.1 ± 9.74
Female, *n* (%)	130 (43.0)	50 (48.5)	35 (34.7)	45 (45.9)	46 (44.7)	176 (43.5)
Race, *n* (%)						
White	265 (87.7)	95 (92.2)	87 (86.1)	83 (84.7)	88 (85.4)	353 (87.2)
Black/African American	16 (5.3)	5 (4.9)	6 (5.9)	5 (5.1)	6 (5.8)	22 (5.4)
Native Hawaiian/Pacific Islander	0	0	0	0	1 (1.0)	1 (0.2)
Asian	9 (3.0)	1 (1.0)	3 (3.0)	5 (5.1)	5 (4.9)	14 (3.5)
American Indian/Alaska Native	2 (0.7)	0	1 (1.0)	1 (1.0)	0	2 (0.5)
Other	10 (3.3)	2 (1.9)	4 (4.0)	4 (4.1)	3 (2.9)	13 (2.3)
Mean ± SD weight, lb	183.3 ± 40.84	186.1 ± 40.97	179.8 ± 37.61	184.0 ± 43.94	181.2 ± 44.47	182.8 ± 41.75
Mean ± SD BMI, kg/m^2^	28.0 ± 5.31	28.5 ± 5.31	27.1 ± 5.01	28.3 ± 5.54	27.5 ± 5.22	27.9 ± 5.28
Mean ± SD ADHD-RS-IV-TS	40.3 ± 5.57	39.9 ± 5.20	40.9 ± 5.57	40.2 ± 5.95	40.2 ± 5.41	ND
ADHD subtype, *n* (%)						
Inattentive	55 (18.2)	17 (16.5)	19 (18.8)	19 (19.4)	20 (19.4)	75 (18.5)
Hyperactive/impulsive	3 (1.0)	0	2 (2.0)	1 (1.0)	0	3 (0.7)
Combined	244 (80.8)	86 (83.5)	80 (79.2)	78 (79.6)	83 (80.6)	327 (80.3)

^a^ Individual dose data are not available for the dose-optimization study.

^b^ SHP465 MAS, *n* = 135; all, *n* = 267.

^c^ Based on the randomized safety population at baseline (SHP465 MAS, *n* = 136; PBO, *n* = 135).

ADHD, attention-deficit/hyperactivity disorder; ADHD-RS-IV-TS, ADHD-Rating Scale, 4th Edition total score; BMI, body mass index; MAS, mixed amphetamine salts; ND, not determined; PBO, placebo; SD, standard deviation.

### Responder analyses

**FIG. 1.**
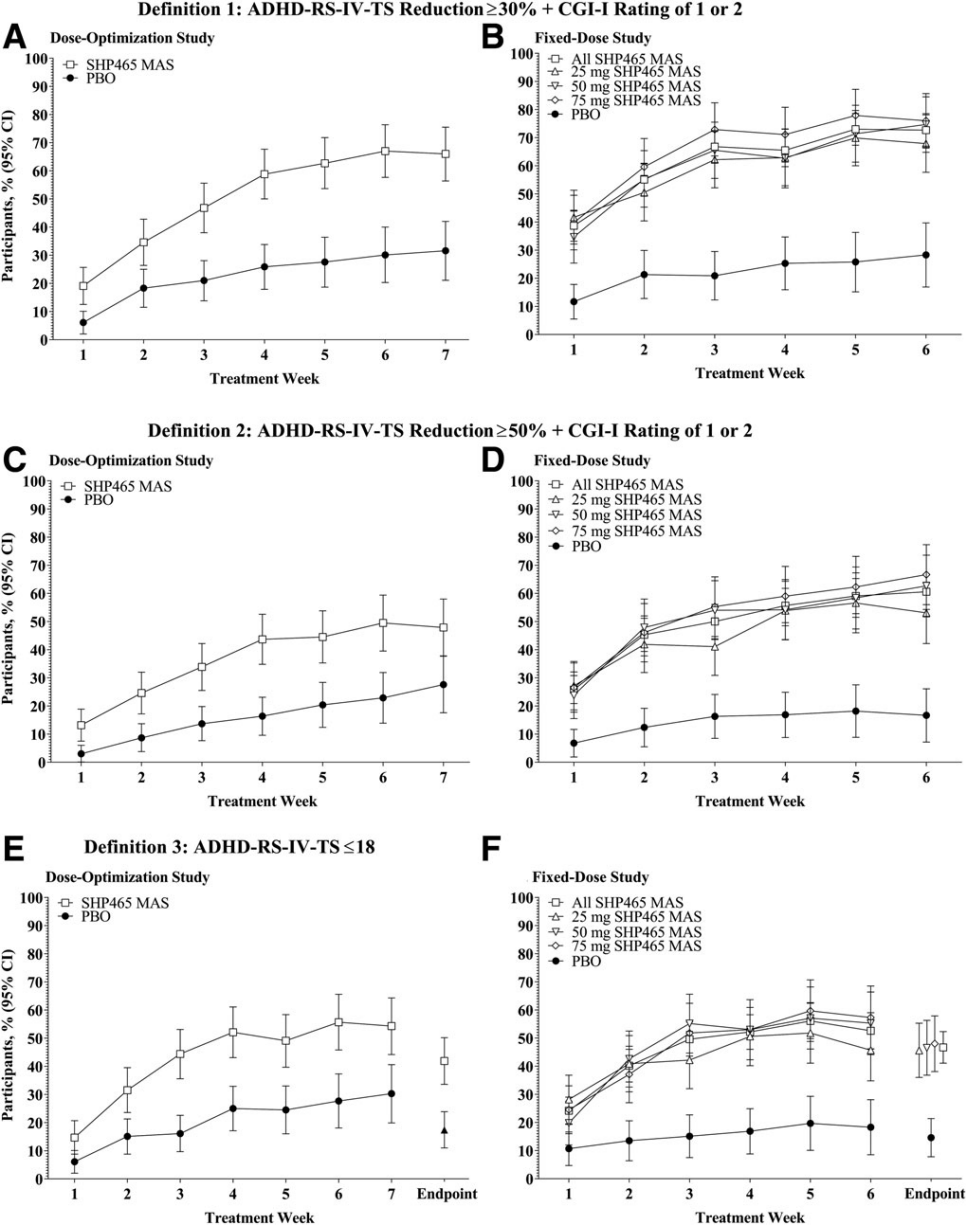
Percentage of participants meeting response criteria by study visit [dose-optimization study **(A**, **C**, **E)**; fixed-dose study **(B**, **D**, **F)]**, in the intent-to-treat population; participants with missing or invalid postbaseline data were excluded. ADHD-RS-IV-TS, ADHD-Rating Scale, 4th Edition total score; CGI-I, Clinical Global Impressions-Improvement; MAS, mixed amphetamine salts; PBO, placebo.

In both studies, numerically greater percentages of participants who received SHP465 MAS versus PBO met treatment response criteria for all response definitions (Fig. 1). The percentages (95% confidence interval [CI]) of participants with a ≥30% ADHD-RS-IV-TS reduction + CGI-I rating of 1 or 2 (definition 1) at the final treatment week were 66.0% (56.4–75.5) in the SHP465 MAS group and 31.6% (21.1–42.0) in the PBO group in the dose-optimization study (Fig. 1A) and were 72.7% (67.0–78.5) in the overall SHP465 MAS group (67.9% [57.7–78.1] for 25 mg, 74.7% [64.8–84.5] for 50 mg, and 76.0% [66.3–85.7] for 75 mg) and 28.3% (16.9–39.7) in the PBO group in the fixed-dose study (Fig. 1B).

The percentages (95% CI) of participants with a ≥50% ADHD-RS-IV-TS reduction + CGI-I rating of 1 or 2 (definition 2) at the final treatment week were 47.9% (37.8–58.0) in the SHP465 MAS group and 27.6% (17.6–37.7) in the PBO group in the dose-optimization study (Fig. 1C), and were 60.6% (54.3–66.9) in the overall SHP465 MAS group (53.1% [42.2–64.0] for 25 mg, 62.7% [51.7–73.6] for 50 mg, and 66.7% [56.0–77.3] for 75 mg) and 16.7% (7.2–26.1) in the PBO group in the fixed-dose study (Fig. 1D).

**FIG. 2.**
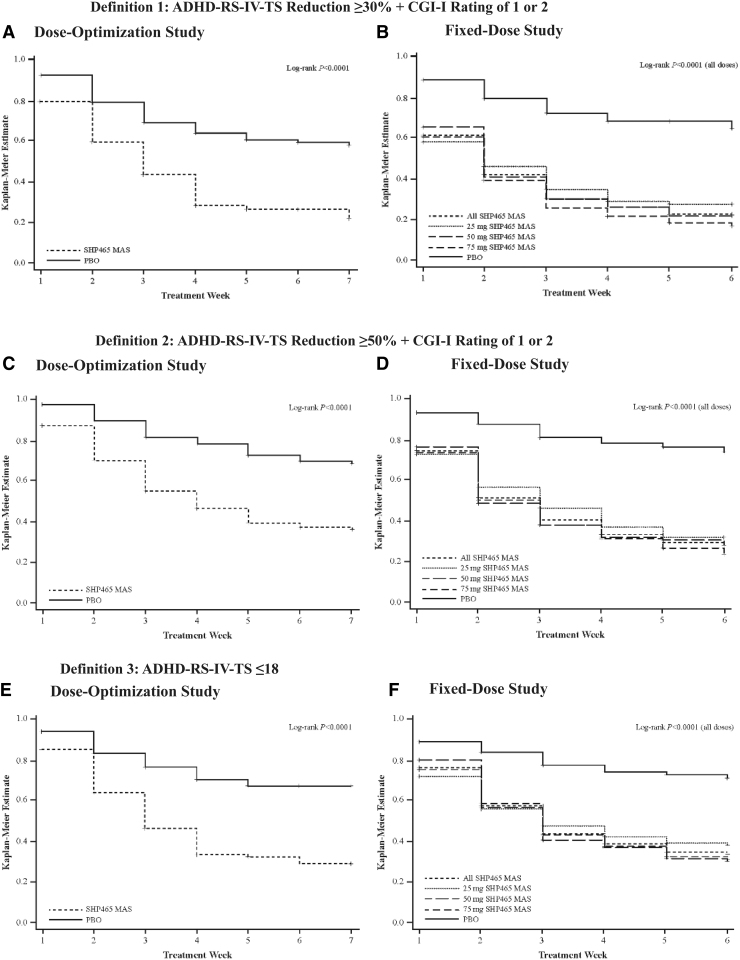
Kaplan–Meier analysis of time to response [dose-optimization study **(A**, **C**, **E)**; fixed-dose study **(B**, **D, F)**] in the intent-to-treat population; participants with missing or invalid postbaseline data were excluded. ADHD-RS-IV-TS, ADHD-Rating Scale, 4th Edition total score; CGI-I, Clinical Global Impressions-Improvement; MAS, mixed amphetamine salts; PBO, placebo.

The percentages (95% CI) of participants with ADHD-RS-IV-TS ≤18 (definition 3) at week 7 and endpoint, respectively, were 54.3% (44.2–64.3) and 41.9% (33.6–50.2) in the SHP465 MAS group, and 30.3% (19.9–40.6) and 17.4% (11.0–23.9) in the PBO group in the dose-optimization study (Fig. 1E). The percentages (95% CI) of participants with ADHD-RS-IV-TS ≤18 at week 6 and endpoint, respectively, were 52.6% (46.2–59.0) and 46.7% (41.1–52.3) in the overall SHP465 MAS group (45.7% [34.8–56.5] and 45.6% [36.0–55.3] for 25 mg, 55.3% [44.1–66.4] and 46.5% [36.8–56.3] for 50 mg, and 57.3% [46.1–68.5] and 48.0% [38.1–57.9] for 75 mg), and 18.3% (8.5–28.1) and 14.6% (7.8–21.4) in the PBO group in the fixed-dose study (Fig. 1F). In both studies, the Kaplan–Meier analyses indicated that time to response favored SHP465 MAS over PBO for all response criteria (all nominal log-rank *p* < 0.0001; Fig. 2).

### Remission analyses

**FIG. 3.**
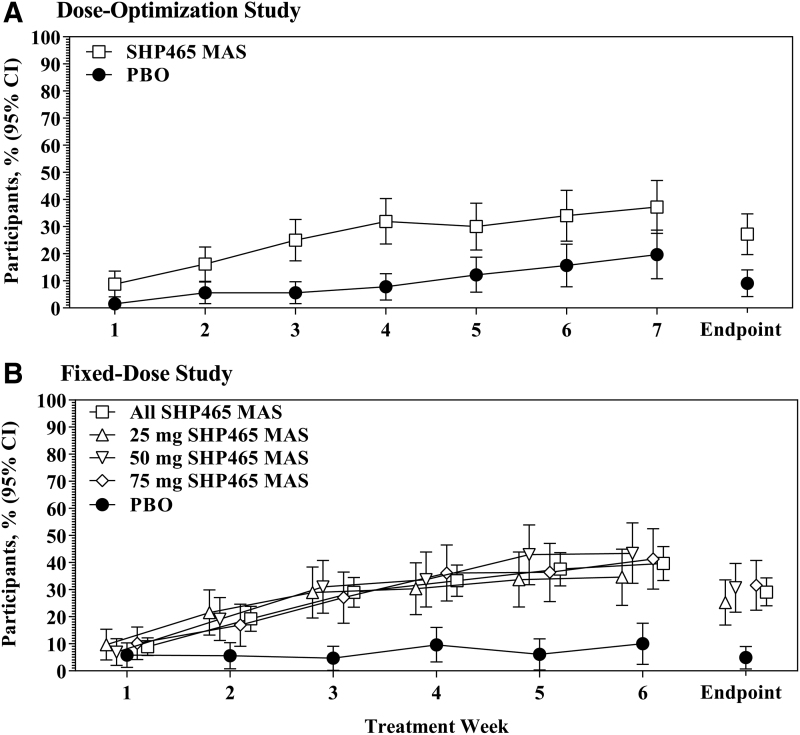
Percentage of participants meeting the remission criterion by visit [dose-optimization study **(A)**; fixed-dose study **(B)**] in the intent-to-treat population; participants with missing or invalid postbaseline data were excluded. MAS, mixed amphetamine salts; PBO, placebo.

**FIG. 4.**
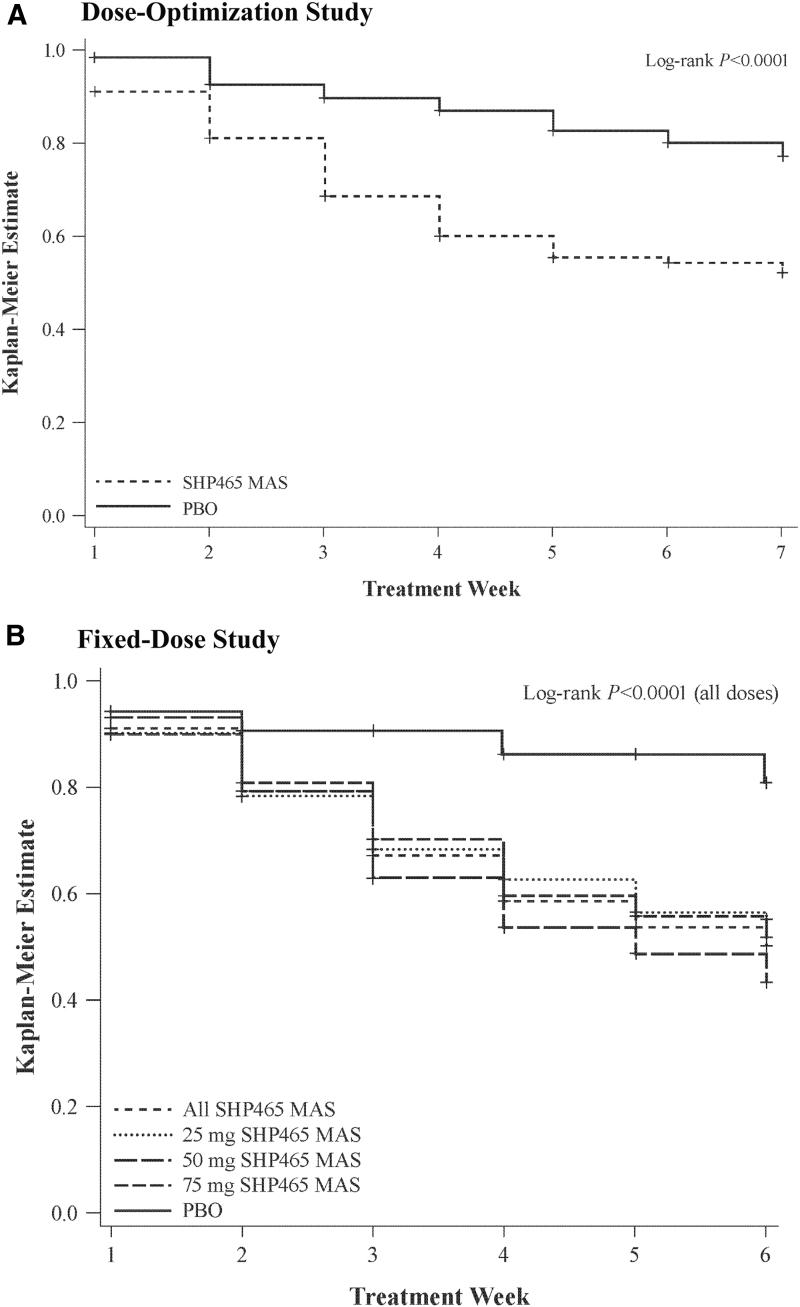
Kaplan–Meier analysis of time to remission [dose-optimization study **(A)**; fixed-dose study **(B)**] in the intent-to-treat population; participants with missing or invalid postbaseline data were excluded. MAS, mixed amphetamine salts; PBO, placebo.

In both studies, numerically greater percentages of participants met the remission criterion with SHP465 MAS than PBO group (Fig. 3). In the dose-optimization study, the percentages (95% CI) of participants meeting the remission criterion were 37.2% (27.5–47.0) at week 7 and 27.2% (19.7–34.7) at endpoint in the SHP465 MAS group, and were 19.7% (10.8–28.7) at week 7 and 9.1% (4.2–14.0) at endpoint in the PBO group (Fig. 3A). In the fixed-dose study, the percentages (95% CI) of participants meeting the remission criterion in the SHP465 MAS group were 39.7% (33.4–45.9) at week 6 (34.6% [24.2–44.9] for 25 mg, 43.4% [32.3–54.6] for 50 mg, and 41.3% [30.2–52.5] for 75 mg) and 29.1% (24.0–34.3) at endpoint (25.2% [16.9–33.6] for 25 mg, 30.7% [21.7–39.7] for 50 mg, and 31.6% [22.4–40.8] for 75 mg), and in the PBO group the corresponding values were 10.0% (2.4–17.6) at week 6 and 4.9% (0.7–9.0) at endpoint (Fig. 3B). The Kaplan–Meier analyses indicated that time to remission favored SHP465 MAS over PBO in both studies (all nominal log-rank *p* < 0.0001; Fig. 4A, B).

Remission rates at endpoint based on sex, age, and baseline symptom count are summarized in Table 2. In both studies, numerically greater percentages of participants in the SHP465 MAS group than the PBO group met the remission criterion when assessed as a function of sex, mean age, and mean baseline symptom count. There were no consistent trends across studies indicative of differential remission rates at endpoint based on sex or mean age. Remission rates associated with SHP465 MAS treatment at endpoint in both studies were numerically greater in participants who had baseline symptom counts that were below the group mean compared with participants who had baseline symptom counts that were above the group mean, with the exception of the 75 mg SHP465 treatment group in the fixed-dose study.

**Table 2.** Percentage of Participants Meeting the Remission Criterion at Endpoint by Sex, Age, and Baseline Symptom Count, Intent-to-Treat Population

	Dose-optimization study^a^									
	All SHP465 MAS								PBO	
	n	% (95% CI)							n	% (95% CI)
Sex										
Men	69	21.7 (12.0–31.5)							67	11.9 (4.2–19.7)
Women	67	32.8 (21.6–44.1)							65	6.2 (0.3–12.0)
Mean age, years										
<36.5	65	26.2 (15.5–36.8)							54	11.1 (2.7–19.5)
≥36.5	71	28.2 (17.7–38.6)							78	7.7 (1.8–13.6)
Mean baseline										
ADHD-RS-IV-TS										
<36	72	33.3 (22.4–44.2)							69	10.1 (3.0–17.3)
≥36	64	20.3 (10.5–30.2)							63	7.9 (1.3–14.6)
	*Fixed-dose study*									
	*All SHP465 MAS*			*25 mg*		*50 mg*		*75 mg*		*PBO*
	n	*% (95% CI)*	n	*% (95% CI)*	n	*% (95% CI)*	n	*% (95% CI)*	n	*% (95% CI)*
Sex										
Men	172	29.7 (22.8–36.5)	53	34.0 (21.2–46.7)	66	27.3 (16.5–38.0)	53	28.3 (16.2–40.4)	57	3.5 (0.0–8.3)
Women	130	28.5 (20.7–36.2)	50	16.0 (5.8–26.2)	35	37.1 (21.1–53.2)	45	35.6 (21.6–49.5)	46	6.5 (0.0–13.7)
Mean age, years										
<37.1	146	29.5 (22.1–36.8)	45	26.7 (13.7–39.6)	51	33.3 (20.4–46.3)	50	28.0 (15.6–40.4)	58	5.2 (0.0–10.9)
≥37.1	156	28.8 (21.7–36.0)	58	24.1 (13.1–35.2)	50	28.0 (15.6–40.4)	48	35.4 (21.9–48.9)	45	4.4 (0.0–10.5)
Mean baseline										
ADHD-RS-IV-TS										
<40	140	32.1 (24.4–39.9)	50	32.0 (19.1–44.9)	39	33.3 (18.5–48.1)	51	31.4 (18.6–44.1)	48	4.2 (0.0–9.8)
≥40	162	26.5 (19.7–33.3)	53	18.9 (8.3–29.4)	62	29.0 (17.7–40.3)	47	31.9 (18.6–45.2)	55	5.5 (0.0–11.5)

^a^ Individual dose data are not available for the dose-optimization study.

ADHD-RS-IV-TS, ADHD-Rating Scale, 4th Edition total score; MAS, mixed amphetamine salts; PBO, placebo; CI, confidence interval.

## Discussion

These *post hoc* analyses of data from two previously published SHP465 MAS clinical studies in adults diagnosed with ADHD (Spencer et al. 2008; Frick et al. 2020) indicate that ADHD symptom improvement with SHP465 MAS is associated with a substantial proportion of study participants meeting criteria for clinically relevant treatment response and remission of ADHD symptoms. The key findings are that greater percentages of participants treated with SHP465 MAS than with PBO met criteria for response and remission at the final treatment week and/or study endpoint, with time to response and remission nominally favoring SHP465 MAS over PBO. Separation from PBO was observed 1–3 weeks after the initiation of treatment. Furthermore, remission rates within each study were roughly comparable when examined based on sex and age, but tended to be numerically greater in participants with lower versus higher baseline symptom counts.

In the current analyses, SHP465 MAS treatment response was examined using variations of definitions described in the published literature (Adler et al. 2009, 2014; Dickson et al. 2011; Jain et al. 2011; Mattingly et al. 2013; Dittmann et al. 2014; Weiss et al. 2018). Across definitions, response rates at the final treatment visit ranged from 47.9% to 66.0% in the SHP465 MAS group in the dose-optimization study and from 52.6% to 72.7% in the overall SHP465 MAS group in the fixed-dose study. The observed response rates with SHP465 MAS were ∼1.7 to 3.6 times greater than the rates observed with PBO and further support the efficacy of SHP465 MAS versus PBO. Not unexpectedly, increasing the stringency of the response criteria was generally associated with a decrease in response rate.

The magnitude of the response rates for SHP465 MAS observed in these studies is within the range observed for other long-acting psychostimulants in adults diagnosed with ADHD (Adler et al. 2009, 2014; Mattingly et al. 2013). In a 4-week, forced-dose study of lisdexamfetamine dimesylate (LDX) in adults diagnosed with ADHD, 67%–70.9% of LDX-treated participants met response criteria (ADHD-RS-IV-TS reductions ≥30% + CGI-I rating of 1 or 2) at any time during the study compared with 37.1% of PBO-treated participants (Mattingly et al. 2013). In a 7-week study of osmotic controlled-release MPH (OROS-MPH) in adults diagnosed with ADHD, 36.9% of OROS-MPH-treated participants and 20.9% of PBO-treated participants met response criteria (>30% AISRS score reduction + CGI-I rating of 1 or 2) at the final visit (Adler et al. 2009).

To the best of our knowledge, there are no published data reporting response rates for MAS extended-release in adults diagnosed with ADHD. However, in a small crossover study of MAS IR and LDX, response rates (defined as ADHD-RS with adult prompt TS reductions ≥30%) were 76.2% (16/21) and 82.6% (19/23) with MAS IR and LDX, respectively, after 5 weeks of treatment (Adler et al. 2014). Taken together, these data suggest that the observed response rates in adults treated with SHP465 MAS are generally comparable with other long-acting psychostimulants and with MAS IR. However, comparisons between these studies are limited by differences in study design (e.g., duration of exposure and ADHD severity at baseline) and the definitions of response used across studies.

The definition of remission in the current analyses, an ADHD-RS-IV-TS ≤12, was more conservative than definitions used in previously published analyses (Dickson et al. 2011; Jain et al. 2011; Mattingly et al. 2013; Goodman et al. 2017; Weiss et al. 2018, 2019). The conservative nature of this remission definition is further emphasized by the fact that an ADHD-RS-IV-TS of 12 corresponds to one-half of the AISRS total score that Silverstein et al. (2018) observed in 95% of adult ADHD controls. As there are 18 items in the ADHD-RS-IV, a total score ≤12 indicates that most items were likely scored as 0 or 1, which is indicative of a low level of ADHD symptoms. Using this definition, remission rates at the final visit and endpoint, respectively, were 37.2% and 27.2% with SHP465 MAS in the dose-optimization study, and were 39.7% and 29.1% in the overall SHP465 MAS group in the fixed-dose study. These observed remission rates were 1.9–3.0 times greater than PBO in the dose-optimization study and 4.0–5.9 times greater than PBO in the fixed-dose study.

We are not aware of other studies to date that have used a similarly conservative definition for remission. However, it is not surprising to note that the use of a more conservative definition in the current analyses resulted in slightly lower remission rates than have been reported for other psychostimulant formulations (Mattingly et al. 2013; Goodman et al. 2017). In one published report that defined remission in adults with ADHD as an ADHD-RS-IV-TS ≤18 at any time during the study, remission was achieved by 40.2%–51.7% of LDX-treated participants and 16.1% of PBO-treated participants (Mattingly et al. 2013). In another study of adults diagnosed with ADHD, remission rates (defined as AISRS scores <18) were 45.0% with OROS-MPH and 30.8% with PBO (Goodman et al. 2017).

In the current analyses, there were no apparent differences in remission rates based on sex or age. However, in both studies, remission rates tended to be numerically greater among participants with baseline ADHD symptom counts that were below the group mean compared with participants with baseline ADHD symptom counts that were above the group mean. These findings suggest that lower ADHD symptom severity was associated with an increased probability of achieving remission. This may be because having a lower baseline ADHD-RS-IV-TS means a lesser magnitude reduction in ADHD-RS-IV-TS is required to meet the remission criterion of ADHD-RS-IV-TS ≤12. However, it is important to note that mean ADHD-RS-IV-TS at baseline tended to be higher in the fixed-dose study relative to the dose-optimization study (∼40 points vs. 36 points), but remission rates tended to be lower in the dose-optimization study relative to the fixed-dose study (final visit, 37.2% vs. 39.7%; endpoint, 27.2% vs. 29.1%). The reasons for the discrepancy between the within-study analysis of remission based on baseline symptom count and the comparison of remission rates and mean ADHD-RS-IV-TS at baseline between studies are not known.

Times to response and remission favored SHP465 MAS over PBO in both studies, with separation from PBO being observed after 1–2 weeks of treatment for response and after 2–3 weeks of treatment for remission. These findings are consistent with those of previous studies of LDX (Mattingly et al. 2013) and OROS-MPH (Adler et al. 2009), which also show early separation from PBO. Adler et al. (2009) reported that the percentage of study participants meeting criteria for treatment response, defined as a ≥30% reduction in AISRS score plus a CGI-I score of 1 or 2, was significantly greater with OROS-MPH compared with PBO after the first dose titration (∼7 days). Furthermore, Mattingly et al. (2013) reported that the median time to clinical response, defined as a ≥30% ADHD-RS-IV-TS reduction and a CGI-I rating of 1 or 2, with LDX was 15 days.

Although the longer delay in meeting the specified remission criterion compared with the response criteria could be related to the study design (e.g., weekly intervals between assessments, differences in the criteria for response and remission), it is more likely related to the fact that the remission criterion is more stringent than the response criteria. As such, attaining remission (i.e., an ADHD-RS-IV-TS ≤12) likely requires a higher magnitude score reduction from baseline than does attaining ≤30% or ≤50% reductions in ADHD-RS-IV-TS. The ∼1-week delay in meeting the remission criterion may represent the additional time required to attain these higher magnitude score reductions. Furthermore, while some ADHD symptoms may remit quickly after the initiation of stimulant pharmacotherapy, others may take longer to respond to treatment. For example, in regard to misplacing items, even though attention and focus may improve rapidly, it may take some time for individuals with ADHD to improve their organizational skills to the point that items are no longer misplaced.

The concepts of treatment response and symptomatic remission explored in these analyses are both important from a clinical perspective. Treatment response criteria can be used by clinicians during the titration of pharmacotherapy to ensure effective doses are utilized. Remission criteria can be used to determine if ADHD symptom levels are reduced to a level associated with a lack of ADHD-associated impairment. Importantly, the definitions of response and remission utilized in these analyses are indicative of clinically relevant reductions in ADHD symptoms. Based on analyses conducted by Goodman et al. (2010), ADHD-RS-IV-TS reductions of 25%–30% correspond to a one-point change on the CGI-I and 50%–60% reductions are needed to achieve a CGI-I rating of 2 (i.e., a rating of much improved). Furthermore, ADHD-RS-IV-TS ≤18 or ≤12 are generally indicative of no substantive ADHD symptoms because these scores suggest that most item scores do not exceed 1 (i.e., a rating of “sometimes”) in regard to recent ADHD-related behaviors.

When considering these data, several limitations should be considered. First, the study was not powered for these *post hoc* analyses. Therefore, all reported *p* values are nominal and descriptive. Second, the study population was predominantly white and had the combined ADHD subtype. Therefore, it is not known whether these data would generalize to a more heterogeneous population of adults with ADHD. Finally, data are not currently available linking the treatment response and remission criteria described in this report to improvement in specific aspects of functioning. Further research in this area is recommended to provide additional insight into the clinical and functional meaningfulness of the criteria used in these analyses.

## Conclusions

These *post hoc* analyses demonstrate that SHP465 MAS was associated with response and remission rates, respectively, that were 1.7–3.6 times and 1.9–5.9 times greater than PBO. Importantly, even though a conservative definition of remission was used, a substantial percentage of participants (∼30%–40%) met remission criteria at the final visit and/or study endpoint. Times to response and remission also nominally favored SHP465 MAS over PBO, with separation from PBO being observed after 1–2 weeks for response and after 2–3 weeks for remission. Taken together, these findings further indicate that ADHD symptom improvement with SHP465 MAS is associated with a substantial proportion of study participants meeting criteria for clinically relevant treatment response and remission of ADHD symptoms.

## Clinical Significance

SHP465 MAS extended-release is a once-daily oral psychostimulant approved for the treatment of ADHD in individuals aged ≥13 years. In two studies, SHP465 MAS significantly reduced ADHD symptoms versus PBO, as measured by ADHD-Rating Scale, 4th Edition total score in adults diagnosed with ADHD. These *post hoc* analyses demonstrate that rates of treatment response and remission with SHP465 MAS exceed those observed with PBO, and that the time to response and remission nominally favors SHP465 MAS over PBO, with separation from PBO being observed after 1–2 weeks of treatment for response and after 2–3 weeks of treatment for remission. These findings further emphasize the efficacy of SHP465 MAS versus PBO in reducing the symptoms of ADHD in adults.
